# Starvation in Enteric Fever

**Published:** 1890-10-11

**Authors:** 


### The Practitioner's Mirror and Retrospect.

*[The Editor will be glad to receive offers of co-operation and contributions from members of the profession. There is no doubt that much valuable material full of interest and instruction is at present lost to science. This is largely so in the case of (1) the younger and less-known members of the hospital staff (2) those connected with cottage hospitals, and (3) the great body of medical practitioners not attached to any institution. The co-operation of all is cordially invited to enable the Editor to make* The Hospital *of the utmost possible use and value to the profession. Specialists willing to review books on their special subjects are invited to communicate this fact at once. All letters should be addressed to* The Editor, The Lodge, Porchester Square, London, W.]

### MEDICINE.

### STARVATION IN ENTERIC FEVER.

Dr. Licovish maintains that he has for six years met with unusual success in the treatment of typhoid fever by *absolute* rest in the recumbent posture and by withholding all food for several days from the commencement of the attack. Sometimes he allows very small quantities of milk, not exceeding 4 oz. daily, in others he gives nothing but water. After this has been persisted in for four, five, or six days the patients, who commonly loathe all nourishment, usually complain of hunger, when he begins to gratify the returning appetite very cautiously and gradually with small quantities of such light nourishment as milk, beef tea, peptonised foods, or farinacea, as the patient may choose, and convalescence is soon established. He believes that through the physiological or functional rest thus obtained the effete matters are eliminated, and not, as they would otherwise be, increased by the products of metabolism of the food; the catarrhal condition of the stomach and bowels subsides, and consequently the temperature either falls at once or does so after having remained stationary for a few days longer. In fact, the fever may be said to be aborted, though it will return if the least indiscretion in the way of a premature or too liberal administration of nourishment be permitted. He has thus treated no fewer than six hundred cases. The appetite created by the deprivation of food enables the patient to utilise it, when it is at length given, in a way that he would not had it been supplied from the first. But that he may be able to bear this starvation the strictest rest in the recumbent posture is imperative, as any effort or raising of the head or trunk may be followed by syncope.

We recollect reading some years ago a communication from an English medical man, who, having been attacked with typhoid of a severe type, absolutely refused all nourishment for over a week if not for a fortnight, with results gratifying to himself and not a little surprising to his friends, who had feared he would soon fall a victim to his obstinacy.

It is, in our opinion, certain that in all acute febrile diseases, in which the blood is laden with the products of abnormally accelerated albuminous metabolism of the tissues, as indicated by the loss of weight and the excessive elimination of urea and urates by the kidneys, the addition to this mass of effete materials by a highly nitrogenous diet, must be attended with grave results, and we have invariably prescribed a much larger proportion of carbohydrates in the form of arrowroot, cornflour, barley water, etc., than is customary, since the metabolism of these leads to the production of carbonic acid and water only, which are easily eliminated, and does not impose any work on the already over-taxed kidneys.

